# Targeting P-selectin blocks neuroblastoma growth

**DOI:** 10.18632/oncotarget.21364

**Published:** 2017-09-28

**Authors:** Riitta Nolo, Shelley Herbrich, Arvind Rao, Patrick Zweidler-McKay, Sankaranarayanan Kannan, Vidya Gopalakrishnan

**Affiliations:** ^1^ Departments of Pediatrics, M.D. Anderson Cancer Center, Houston, TX, USA; ^2^ Molecular and Cellular Oncology, M.D. Anderson Cancer Center, Houston, TX, USA; ^3^ Bioinformatics and Computational Biology, M.D. Anderson Cancer Center, Houston, TX, USA; ^4^ Center for Cancer Epigenetics, M.D. Anderson Cancer Center, Houston, TX, USA; ^5^ Brain Tumor Center, University of Texas, M.D. Anderson Cancer Center, Houston, TX, USA; ^6^ ImmunoGen Inc., Waltham, MA, USA

**Keywords:** neuroblastoma, P-selectin, selectin inhibition, CyTOF, tumor heterogeneity

## Abstract

Selectins and their ligands have been implicated in tumor growth and progression in carcinomas, but their role in neuroblastoma has not been systematically examined. In the current study we evaluated L-, P- and E-selectin binding to neuroblastoma cells and the expression of some of their known ligands, namely CD44, CD24 and P-selectin glycoprotein ligand-1 (PSGL-1). Genetic loss of PSGL-1 or CD24 and pharmacological inhibition of P-selectin reduced P-selectin binding to neuroblastoma cells i*n vitro*. Targeting P-selectin using specific antibodies promoted a significant reduction in the growth of neuroblastoma tumors *in vivo*. In mechanistic studies binding of P-selectin to neuroblastoma cells activated Src and several other pro-survival kinases such as ERK1, AKT, FAK and p38. Interestingly, comparative mass single cell cytometry (CyTOF) analyses revealed considerable intra- and inter-cell line heterogeneity with respect to response to P-selectin binding. Additionally, the downstream response to all selectins showed general similarity. Our findings reported here not only provide pre-clinical evidence in support of therapeutic targeting of P-selectin, but also highlight the heterogeneity in response of tumor cells to P-selectin binding. These observations provide the basis for combining P-selectin inhibition with other targeted therapies for neuroblastoma.

## INTRODUCTION

Neuroblastomas are the most common extracranial solid tumors in children and originate from cells of the embryonic neural crest. They account for nearly 7% of all childhood cancers and 15% of pediatric cancer-related deaths, with more than 650 cases diagnosed each year in the US [1, 2]. Survival rates of up to 95% are seen in patients with low-risk neuroblastoma with current standard of care [1, 2]. However, survival of patients with tumor dissemination or other features of high-risk neuroblastoma continues to hover around 40%, in part due to lack of therapeutics that target their refractory disease [1, 2]. Despite improvements in staging and molecular stratification of tumors over the last decade, progress in identifying drivers of tumorigenic behavior and in translating this information to improving clinical outcomes has been slow [3].

Glycoproteins in normal cells mediate a whole range of processes including cell-cell recognition, cell adhesion and modulation of immune cell function, to name a few [4, 5, 6]. CD24, CD44 and P-selectin glycoprotein ligand-1 (PSGL-1) are glycoproteins whose overexpression is associated with poor prognosis in various carcinomas [7, 8, 9]. For example, high expression of CD24 is correlated with tumor progression and metastasis in hepatocellular carcinoma and bladder cancer, and blocking its activity by ɑ-CD24 antibodies in mouse models reduced tumorigenesis [10]. Tumors of neuroectodermal origin such as neuroblastoma are characterized by changes in the landscape of cell surface protein glycosylation [11]. However, the relevance of these changes to neuroblastoma pathogenesis is unclear. In neuroblastoma CD24 is highly expressed in undifferentiated tumors [12] and the expression of CD44 is correlated with better prognosis [13, 14]. PSGL-1 is known to be important for immune cell trafficking and regulation of immune activity but its role in neuroblastoma has not been investigated thus far [6].

The ligands for the above receptors are a separate class of transmembrane adhesion glycoproteins called selectins. They are expressed mainly by leukocytes (L-selectin), platelets (P-selectin) or endothelial cells (P- and E-selectin). Extracellular portions of selectins can be shed, and selectins can also be presented in a soluble form [15]. In many cancers, this correlates with metastasis [15]. In the context of cancer biology all three selectins are considered to be facilitators of metastasis [16]. Roles for E-selectin and P-selectin in metastasis *in vivo* and in lung colonization of cancer cells respectively, have been described [17]. L-selectin deficiency has been shown to cause a decline in metastasis in mice [18, 19]. Selectin-selectin ligand interaction can vary considerably not only due to differences in affinity, but also nuances in cell and context specificities, and potential redundancies in this partnership [20, 21]. P-, E- and L-selectin can all bind CD24 and CD44 [22, 23]. However, P-selectin is the highest affinity ligand for PSGL-1 [24]. Selectins are also known to have ligands other than those described above [25]. For example P-and L-selectins can bind heparin and heparan sulfates [26]. Although selectins have not been studied carefully in the context of neuroblastoma, a previous study showed that neuroblastoma cells can bind to activated platelets and that this could be blocked by ɑ-P-selectin antibodies or treatment of tumor cells with neuraminidase or trypsin [27]. Recent work by Schwankhaus and colleagues showed a variable dependency of neuroblastoma metastasis on selectins [28].

In the current report, we investigated the importance of selectins and their ligands in neuroblastoma. Specifically, we examined their expression, interaction with their ligands and their requirement for neuroblastoma cell survival. We also assessed the effect of blocking signaling through P-selectin on tumor growth *in vivo*. Our studies revealed that the selectin ligand *CD24* expression was higher in neuroblastomas compared to the more differentiated ganglioneuroma and ganglioneuroblastoma samples, whereas the converse was true of *CD44.* However, patients with higher *CD44* and or *CD24* in their tumors exhibited better trend of survival. *SELPLG* (PSGL-1) gene expression was more variable and was not associated with prognostic significance. The expression of all selectins was lower in neuroblastomas compared to the more differentiated tumors. Interestingly, the elevated expression of P-selectin (*SELP*) and E-selectin (*SELE)* was a predictor of poorer survival, whereas similar differences in L-selectin (*SELL*) expression did not have prognostic significance. Because P-selectin expression appeared to be associated with poorer survival, we examined its binding to PSGL-1, CD24 and CD44. These studies revealed the genetic loss of one family member to not significantly alter the binding of exogenously added P-selectin binding, suggesting potential redundancy in utilization of its ligands. However, blocking P-selectin binding promoted a significant reduction in the growth of neuroblastoma tumors *in vivo*. In mechanistic studies we observed a significant level of inter and intra-cell line heterogeneity in response to P-selectin exposure based on comparative CyTOF analysis. Differences were largely noted with respect to activation of pro-survival kinases such as Src, ERK1, AKT, FAK and p38 as well as levels of molecules such as GD2, c-Myc, Survivin, p53, Notch2, Notch3, CD24, CD44 and CD274. Thus, our studies not only underscore the relevance of therapeutic targeting of neuroblastoma surface glycoproteins, but also highlight the substantial heterogeneity in molecular signaling response in tumor cells following binding of glycoproteins such as P-selectin.

## RESULTS

### Selectin and selectin ligand expression in human neuroblastoma samples

**Figure 1:**
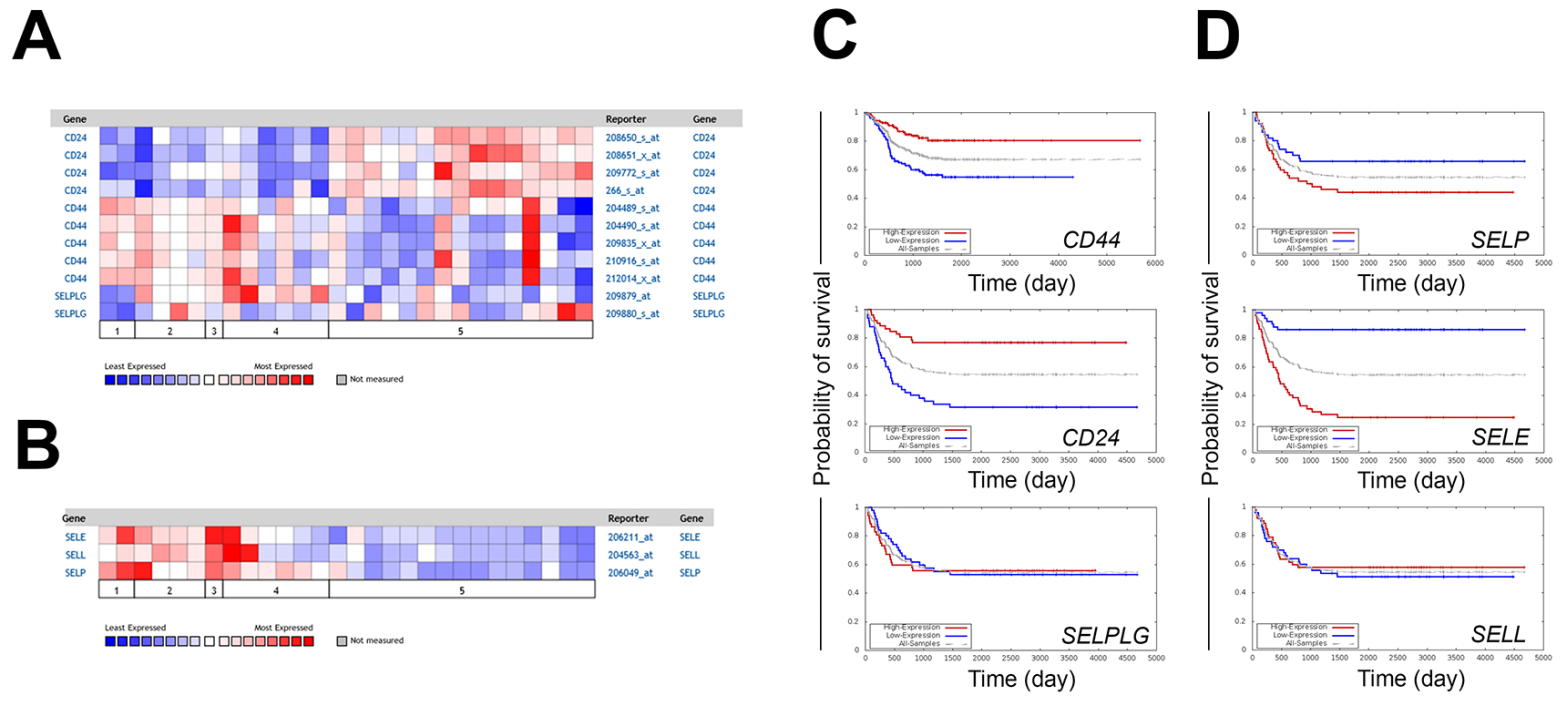
Expression of selectin ligands and selectins in neuroblastoma patients **(A)** Expression of selectin ligand **(B)** and selectin mRNAs in neuroblastoma patient samples. 1 = ganglioneuroblastoma (n=2), 2 = ganglioneuroma (n=4), 3 = mature ganglioneuroma (n=1), 4 = maturing ganglioneuroma (n=6), and 5 = neuroblastoma (n=15). **(C)** Kaplan-Meier curves predicting the patient prognoses based on the expression of *CD44*, *CD24, SELPLG* **(D)** *SELP, SELE* and *SELL* (P-, E- and L-selectin). Probes for *CD44, CD24, SELPLG, SELP, SELE* and *SELL* were A_32_P215002, 266_s_at, 209880_at, 206049_at, 206211_at and 204563_a respectively. N=52/50 in high/low expression patient groups except with CD44 probe n=126/125, respectively. Presented neuroblastoma patient data was extracted using Oncomine (A-B) and Oncogenomics (C-D) databases.

Gene expression of selectins and their ligands and corresponding prognostic information were mined from existing databases (Oncomine^™^, Oncogenomics). *CD24* expression as measured by all four probes was higher in neuroblastomas compared to the more differentiated ganglioneuroma and ganglioneuroblastoma samples (Figure 1A). In contrast, the expression of *CD44* was higher in ganglioneuromas and ganglioneuroblastomas when compared to neuroblastomas and only in one neuroblastoma patient *CD44* was high with all probes (Figure 1A) [29]. The pattern of *SELPLG* (PSGL-1) expression was less definitive and varied depending on specific probes under consideration (Figure 1A) [29]. Expression of all selectins was lower in neuroblastomas compared to the more differentiated tumors (Figure 1B) [29]. Similar trends were seen in different patient cohort data from Janoueix-Lerosey in Oncomine. (not shown). Kaplan-Meier survival curves generated from Oncogenomics-based microarray datasets (5 probes in each) revealed higher expression of both *CD44* and *CD24* to be associated with better survival (Figure 1C). However, differences in the level of *PSGL-1* did not appear to have a consequence with respect to overall survival (Figure 1C) [30, 31]. Higher expression of P-selectin (*SELP*) and E-selectin (*SELE)* was a predictor of poorer survival, whereas similar differences in L-selectin (*SELL*) expression were not associated with prognostic significance (Figure 1D). Selectins each were assayed with only one probe.

### Selectin binding and expression of selectin ligands in neuroblastoma cells

**Figure 2:**
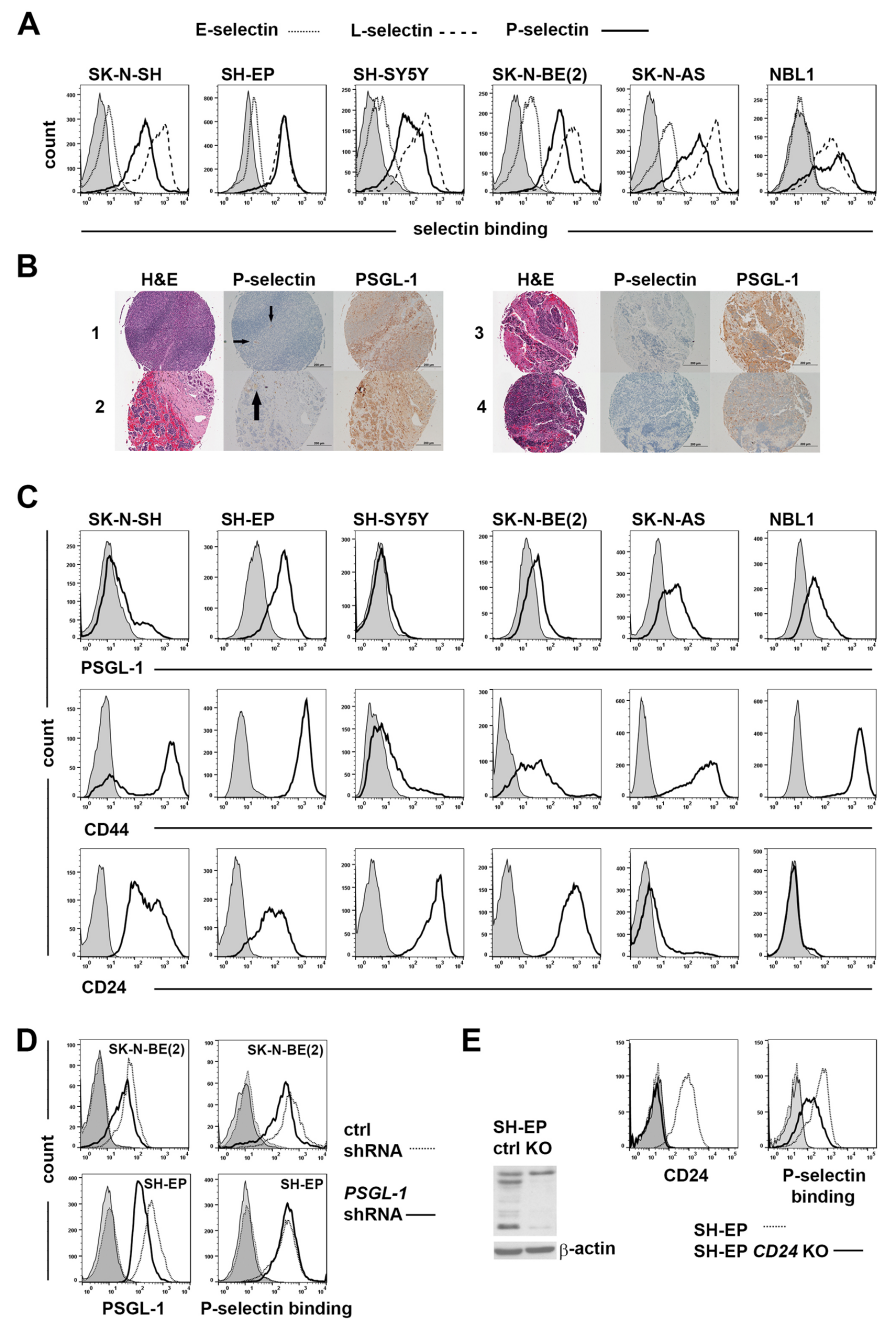
Redundant use of selectin ligands by neuroblastoma cells **(A)** Binding of neuroblastoma cells to selectins. Human IgG Fc and recombinant E-, P- or L-selectin-Fc (10 μg/ml) were incubated each with 300 000 neuroblastoma cells for 30 min and detected with APC ɑ-human IgG Fc antibodies. **(B)** Examples from neuroblastoma tissue microarray showing H&E, P-selectin and PSGL-1 staining. 1) tonsils, a positive control for PSGL-1 staining, small arrows point to endothelial P-selectin staining 2) metastatic neuroblastoma, post treatment, arrow points to P-selectin staining 3) stage 2B neuroblastoma and 4) unfavorable stage 4 neuroblastoma. **(C)** Endogenous expression of PSGL-1, CD44 and CD24 in neuroblastoma cells. PSGL-1 expression was detected by incubating 300 000 cells per sample first with monoclonal ɑ-PSGL-1 and then with APC ɑ-mouse Ig. PE CD44, APC CD24 and isotype controls were incubated each with 500 000 cells. SH-EP and SH-SY5Y cells are sublines of SK-N-SH cells. (D-E) Redundancy of selectin ligand usage in P-selectin binding. **(D)** P-selectin binding is decreased in SK-N-BE(2) cells and unchanged in SH-EP cells due to PSGL-1 silencing. **(E)** Western blot analysis shows the extent of CD24 decrease in SH-EP *CD24* knockout (KO) cells compared to parental SH-EP cells (ctrl). On left flow analysis shows CD24 expression and P-selectin binding of SH-EP *CD24* knockout and control cells. Controls in all flow charts are shaded.

As a first step in assessing the role of selectins in neuroblastoma pathology, a panel of established neuroblastoma cell lines (SK-N-SH, SH-EP, SH-SY5Y, SK-N-BE(2) and SK-N-AS) and a more recently derived patient-derived cell line (NBL1) were studied for their ability to bind various selectins. Cells were incubated in the presence of recombinant P-, E-, or L-selectin-Fc chimeras or human IgG Fc fragment, and efficiency of binding was measured by flow cytometry. Table 1 shows the binding of selectins to neuroblastoma cells as mean fluorescence intensity (MFI) ratios of sample vs control. As shown in Figure 2A and Table 1, all cells bound L- and P-selectins. However, binding to E-selectin was seen to be at a very modest level (Figure 2A and Table 1). Since L-selectin binding is not associated with prognostic significance in patient tumors (Figure 1D) and E-selectin binding to neuroblastoma cells in our panel was low, we focused our investigations on P-selectin levels and binding in further studies. Immunohistochemical (IHC) analysis of tumor (n=47) microarrays showed P-selectin staining in endothelial cells and also in some tumor samples (5 out of 47) with a focal staining pattern (Figure 2B and Supplementary Figure 1). In comparison, PSGL-1 - a known P-selectin ligand was expressed in over 90% samples in the TMA (Figure 2B and Supplementary Figure 1). Western analysis of patient samples using the same antibody showed variable PSGL-1 expression (Supplementary Figure 2). Further, flow cytometric measurement determined the level of PSGL-1 in neuroblastoma cell lines to be more modest in comparison with CD24 and CD44 (Figure 2C). Noticeably, CD24 and CD44 exhibited a reciprocal relationship in their expression levels in any given cell line (Figure 2C).

**Table 1:** Relative binding of selectins to neuroblastoma cells. Mean fluorescence intensity (MFI) of selectin staining divided by MFI of control (Fc) staining

Cell line	E-selectin	L-selectin	P-selectin
SK-N-SH	1.9	133.3	37.8
SH-EP	1.6	19.1	20.5
SH-SY5Y	1.9	64.9	22.8
NBL1	1	11.5	14.8
SK-N-BE(2)	3	100	42.2
SK-N-AS	3.9	171.5	42.1

Given the almost consistent expression of PSGL-1 in patient samples, we examined its utilization in SK-N-BE(2) and SH-EP cells, despite its modest expression in neuroblastoma cells. Partial silencing of PSGL-1 expression was achieved in both SK-N-BE(2) and SH-EP cells by lentiviral expression of a specific *shRNA* and confirmed by flow cytometry (Figure 2D). P-selectin binding was decreased in SK-N-BE(2) cells (Figure 2D), however, its binding in SH-EP cells was unaffected by PSGL-1 decrease (Figure 2D). These results suggest that PSGL-1 is required for P-selectin binding in SK-N-BE(2) cells but this requirement in SH-EP cells can be overcome by utilization of other P-selectin ligands. The requirement for CD24 was similarly evaluated in SH-EP cells by CRISPR system. Knockout was confirmed by Western blot analysis and flow cytometry (Figure 2E). CD24 loss resulted in a decline in P-selectin binding in SH-EP cells (Figure 2E). CD44 utilization was not evaluated because of its association with good prognosis in neuroblastoma patients.

### Blocking P-selectin decreases neuroblastoma growth

**Figure 3:**
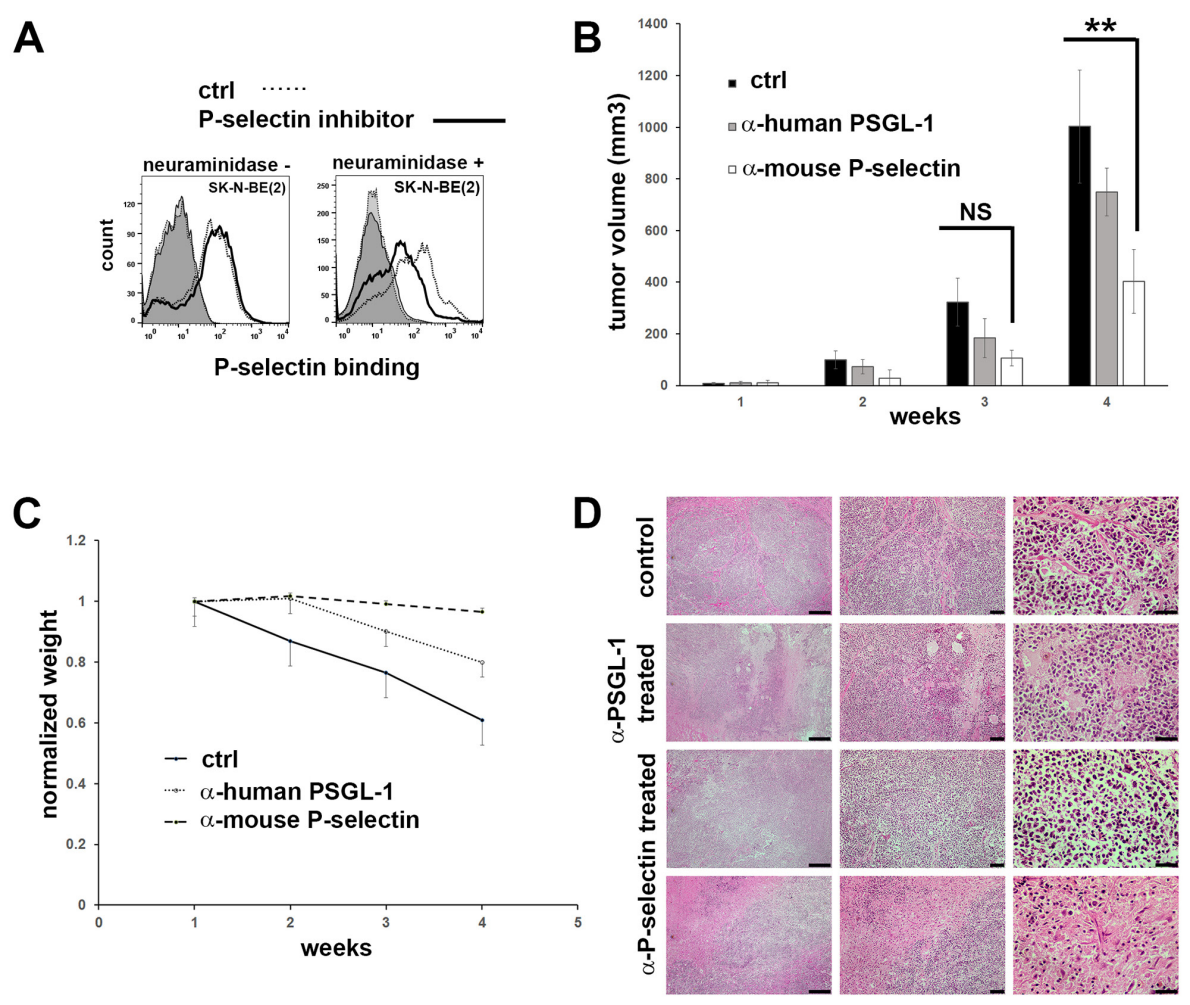
Inhibition of P-selectin binding *in vitro* in SK-N-BE(2) cells and *in vivo* in mice **(A)** P-selectin inhibitor (2-Acetamido-4-fluoro-1,3,6-tri-O-acetyl-2,4-dideoxy-D-glucopyranose) is able partially to block P-selectin binding in SK-N-BE(2) cells, but only if the sialic acid residues are removed by neuraminidase before the drug treatment. After two days in DMSO (control) or in 50 μM P-selectin inhibitor the cells were assayed for their ability to bind Fc (control) and P-selectin-Fc. Binding was detected by APC conjugated ɑ-human IgG Fc antibodies. Controls are shaded. **(B)** Inhibiting mouse P-selectin significantly decreases tumor volume in mice. SK-N-BE(2) cells were injected subcutaneously into mice and after 24 days mice were treated once a week i.v. injections (150 μg/30 g) of mouse IgG2a (control, n=4), ɑ-mouse P-selectin (n=5) or ɑ-human PSGL-1 antibodies (n=4). Mice were treated three weeks. **(C)** ɑ-mouse P-selectin treatment receiving mice maintained normal weight during the experiment. **(D)** H&E staining of mice tumors. Scale bars from left to right are 200, 50 and 20 μm.

To study the requirement of P-selectin in maintaining neuroblastoma growth, SK-N-BE(2) cells were treated with neuraminidase to cleave cell surface carbohydrates and then incubated with the P-selectin inhibitor, 2-acetamido-4-fluoro-1,3,6-tri-O-acetyl-2,4-dideoxy-D-glucopyranose, for 48 hours. A decrease in P-selectin binding to SK-N-BE(2) cells was observed, but only following neuraminidase treatment and blockade of *de novo* synthesized sialylated epitopes by P-selectin inhibitor (Figure 3A). Inhibitory ɑ-PSGL-1 antibodies were also able to partially block P-selectin binding of neuroblastoma cells *in vitro* (data not shown).

To study the effect of P-selectin blockade on tumor cell growth *in vivo*, SK-N-BE(2) cells were injected subcutaneously into immunodeficient NSG-SGM3 mice and tumors were allowed to grow for 24 days. Thereafter, animals received intravenous injections of either mouse IgG2a (control), ɑ-human PSGL-1 or ɑ-mouse P-selectin antibodies at a dose of 150 μg per 30g of body weight, once each week for 4 weeks. Tumor size and weight of animals were constantly monitored. As shown in Figure 3B, animals injected with ɑ-mouse P-selectin antibodies showed a significant decrease (2-tailed *p* value 0.00581) in tumor growth at 4 weeks compared to animals treated with control IgG. Interestingly, ɑ-human PSGL-1 antibodies did not have a statistically significant effect on blocking tumor growth (Figure 3B). This correlated inversely with the weight of animals (Figure 3C). H&E staining of tumor samples from mice from the different treatment groups is shown in comparison with sections from untreated controls (Figure 3D). We also checked the number of innate immune cells by ɑ-rat CD45 staining. Despite the trend of decreasing CD45+ cell counts in ɑ-rat P-selectin treated mice, we found no statistical difference between the treatment groups (Supplementary Figure 3). The above results prove that blocking P-selectin abrogates tumor growth.

### P-selectin activates phosphorylation of Src kinases

**Figure 4:**
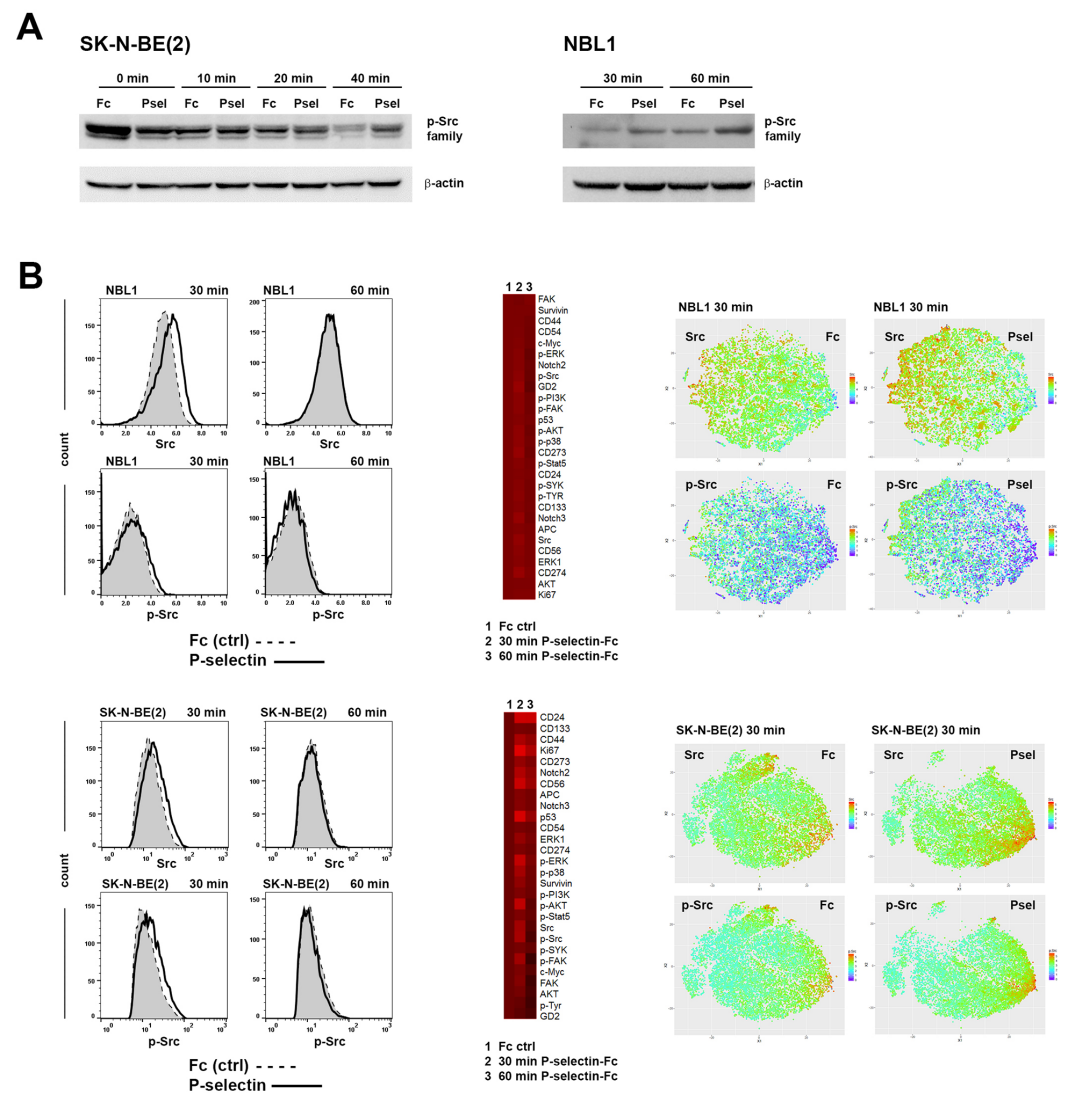
P-selectin binding modulates Src phosphorylation **(A)** Western blot analysis shows P-selectin treatment inducing dynamic changes in p-Src family protein levels in neuroblastoma cells. Equal numbers of dissociated cells were treated various times with control human IgG Fc fragment or recombinant human P-selectin chimera (both 10 μg/ml), lysed and run on gel. 40 μg of protein was loaded per lane. Western blots were incubated with ɑ-p-Src family protein antibodies that detect all phosphorylated Src family members and β-actin was used as a loading control. **(B)** Mass cytometry analysis of Fc and P-selectin treated NBL1 and SK-N-BE(2) cells further confirm the upregulation of Src and p-Src family proteins. Flow charts show the effect of 30 and 60 min Fc (shaded) and P-selectin-Fc treatment on Src and p-Src family expression in NBL1 and SK-N-BE(2) cells. Heat maps in the middle show the relative expression of all measured proteins and viSNE plots on right show in single-cell level the expression of Src and phosphorylated Src family proteins.

Studies from other groups have shown that P-selectin binding to ligand triggers activation of Src kinases prior to changes in cell adhesion [32, 33, 34, 35]. To examine the consequence of P-selectin binding on neuroblastoma cells, SK-N-BE(2) and NBL1 cells were incubated with P-selectin for varying times and cell lysates were subjected to Western blot analyses using rabbit ɑ-p-Src family antibodies that can detect all phosphorylated Src family members. As expected, P-selectin upregulated phosphorylation of Src family of kinases in SK-N-BE(2) and NBL1 cells (Figure 4A).

**Figure 5:**
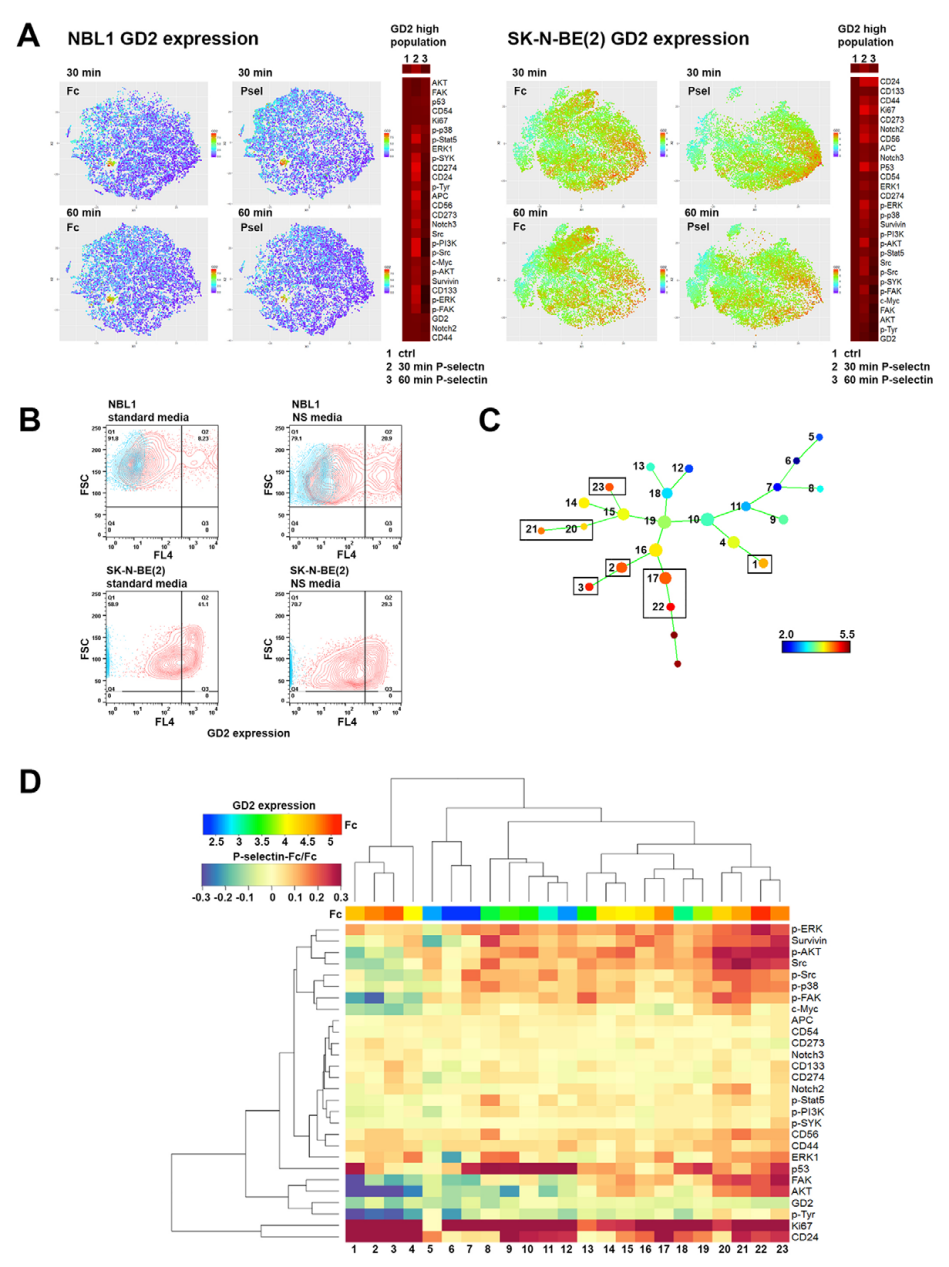
ViSNE and SPADE analysis of P-selectin treated neuroblastoma cells reveals distinct, differentially responding cell populations **(A)** ViSNE plots of GD2 expression reveal in both NBL1 and SK-N-BE(2) cells a GD2 high population that responds to P-selectin treatment. Heat maps on right of each viSNE plot show in gated, highest GD2 expressing cells, the expression levels of all measured proteins in response to 30 and 60 min exposure to P-selectin. **(B)** Culturing NBL1 cells in neurosphere formation allowing conditions (NS media) increases the percentage of GD2 high cell population 2.5 times compared to cells cultured in standard conditions. In contrast, SK-N-BE(2) cells have inherently a higher GD2 expression level and culture in NS media increases the percentage of cells with lesser GD2 staining. Culture time for both cell lines was 15 days. Control is shown in blue in all panels. **(C-D)** The data obtained through mass cytometry from P-selectin treated and control SK-N-BE(2) cells was also analyzed with SPADE. In (C) SPADE tree shows the basal level of GD2 expression in Fc treated SK-N-BE(2) cells. Nodes expressing the highest levels of GD2 are squared. Two populations without numbering represent doubles that sample filtering did not remove and have not been included in heat map. (D) Heat map of the P-selectin/Fc ratios of measured proteins in different SPADE tree populations. Numbers at the bottom correspond to numbering of SPADE tree nodes in C. Notice the control level of GD2 expression on top of the heat map.

We next performed single cell cytometry by time of flight (CyTOF) analyses of NBL1 and SK-N-BE(2) cells to discern differences, if any, in Src phosphorylation in the tumor population. A list of antibodies used for CyTOF analyses is provided in Supplementary Table 1. As seen in Figure 4B, FlowJo and viSNE analyses identified an increase in not only total Src levels, but also in phosphorylation of Src family proteins following 30 min P-selectin treatment. Phosphorylation level was back to control value within 60 min. In addition, P-selectin treatment also caused global increases in phosphorylation of other pro-survival kinases including ERK, p38, AKT and FAK. Selectin ligands CD24 and CD44 were elevated as well as p53, survivin and Notch2, especially in SK-N-BE(2) cells (Figure 4B). Some cell line specific differences were also seen. For example P-selectin increased Ki67 level in SK-N-BE(2) cells but not in NBL1 cells (Figures 4B and 5A). Although an overall elevation in GD2 expression in NBL1 and decrease in SK-N-BE(2) cells following P-selectin treatment for 30 min was noted (Figure 4B), a more careful study revealed GD2-high populations with pronounced changes in response to P-selectin (Figure 5A). Interestingly, the percentage of GD2-expressing NBL1 cells increased during 15 day culture in serum-free media that allows the growth of neural stem cells (Figure 5B). Similar culture conditions decreased the percentage of GD2 high population of SK-N-BE(2) cells (Figure 5B). SPADE analysis of CyTOF data obtained from SK-N-BE(2) cells further revealed and emphasized the heterogeneity in response to P-selectin (Figures 5C and 5D). While GD2 expression itself was either unchanged or declined in response to P-selectin treatment of SK-N-BE(2) cells, distinct subpopulations could be identified based on their molecular signaling signatures. For example, among the GD2-higher population, sub-populations 2-3 were comparatively quiet with respect to pro-survival kinase signaling in contrast to sub-populations 20-23 that exhibit higher level of phosphorylation of ERK, p38, AKT, FAK and Src (Figure 5D). However, cells in sub-population 3 exhibited an increase in the stem cell marker CD133, and were further distinguished by an elevation in Notch2 and CD274 (PD-L1) (Figure 5D). An increase in Notch3 and CD273 was noted in sub-population 2 (Figure 5D). In the GD2-lower cells, sub-population 12 exhibited higher phosphorylation of ERK1 and AKT, and an increase in survivin, p53 and CD24 levels. In contrast, subpopulation 13 was characterized by increased phosphorylation of Src and FAK and elevated c-Myc. Thus, these results highlight the heterogeneous pro-survival signaling response of neuroblastoma cells upon P-selectin-ligand engagement.

### Downstream responses to selectins overlap with some distinct differences

**Figure 6:**
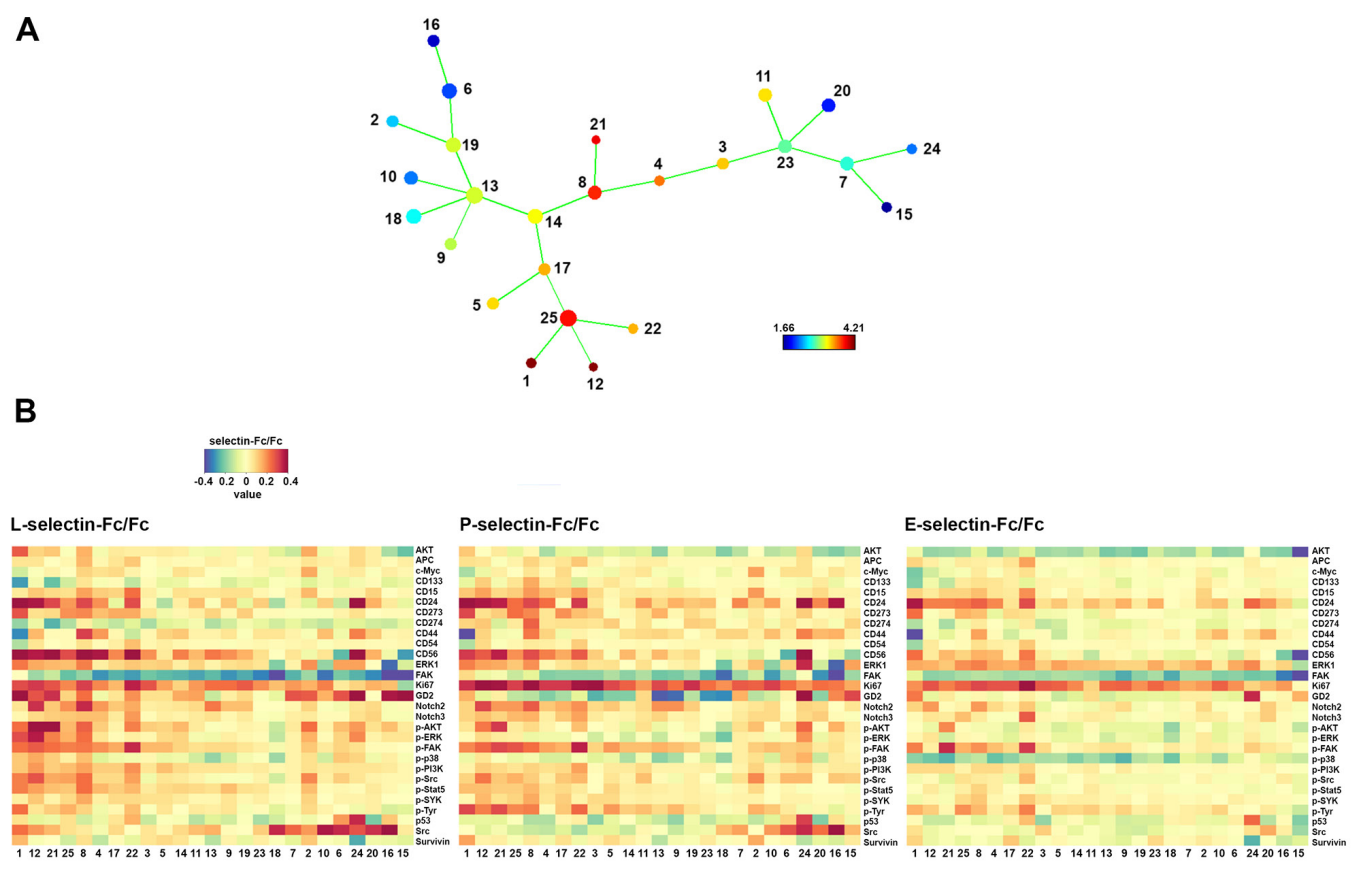
Comparison of SK-N-BE(2) cell response to L-, P- and E-selectin Dissociated cells were treated with control human IgG Fc fragment or recombinant human selectin chimeras (all 10 μg/ml) for 30 min and subjected to staining with metal-tagged antibodies and mass cytometry. **(A)** SPADE tree shows the basal level of GD2 expression in Fc treated cells. **(B)** Heat maps of selectin/Fc ratios in different SPADE tree populations. Numbers at the bottom correspond to numbering of SPADE tree nodes in A and are ordered by the mean basal GD2 expression from highest to lowest beginning from left to right.

Selectins are known functionally to overlap in many cellular contexts [24] and as shown in Figure 2A SK-N-BE(2) cells bind to selectins differently. We compared the response of SK-N-BE(2) cells to L-, P- and E-selectin binding using CyTOF (Figure 6). As stated previously, SK-N-BE(2) cells were incubated for 30 min with selectin-Fc chimeras, subjected to staining and mass cytometry followed by analysis with SPADE (Figure 6A). Generally phosphorylated ERK1, AKT, FAK, STAT5 and Src were demonstrably more upregulated by L- than P-selectin. E-selectin showed the least efficacy in this regard (Figure 6B). Both the intensity of response and the number of responding populations were affected (Figure 6B). On the other hand, a greater upregulation of CD24 was observed in response to P- selectin when compared to L-selectin or E-selectin (Figure 6B). Notable exceptions were CD274, which was specifically upregulated by P-selectin; and CD133 that was upregulated by both P- and E-selectin but not by L-selectin (Figure 6B). However, proteins like survivin and p53 exhibited a similar pattern of response to all three selectins (Figure 6B).

## DISCUSSION

In mammalian epithelial tumors the expression of sialylated and fucosylated glycans like sialyl-Lewis X (sLe^x^) and its structural isomer sialyl-Lewis A (sLe^a^) correlate with poor prognosis due to tumor progression and metastasis [36, 37]. The minimal binding motifs of all selectins consist of above mentioned epitopes and are also carried by all known selectin ligands [15]. In neuroblastoma, selectins have not been studied extensively. To our knowledge, only one *in vivo* study has been performed [28]. According to Schwankhous *et al.* [28] depending on cell line used, metastasis was either dependent on or completely independent of the expression of P- and E-selectins. Selectin dependent SK-N-SH cell line that they used has a similar selectin binding profile as SK-N-BE(2) cells (Figure 2A) used in our *in vitro* and *in vivo* experiments.

The major findings in our study center around the efficacy of P-selectin inhibition against neuroblastoma tumor growth *in vivo* and the heterogeneity of neuroblastoma response to P-selectin exposure *in vitro*. The connection between platelet P-selectin and neuroblastoma cells was described by Stone and Wagner in 1993 [27]. In 1998 Kim *et al.* showed that P-selectin deficiency decreases tumor growth and metastasis of human carcinoma cells in immunodeficient P-selectin null mice [38]. Our findings that preventing P-selectin binding could block neuroblastoma growth in mice are consistent with these observations. We inhibited P-selectin binding by two different approaches, first by blocking PSGL-1 by ɑ-human PSGL1 antibodies and secondly, by inhibiting mouse P-selectin by ɑ-mouse P-selectin antibodies. Inhibiting mouse P-selectin was a more successful approach and significantly reduced the tumor volume suggesting that tumor growth was affected. Even though the response to ɑ-human PSGL-1 was not statistically significant, a trend towards a decrease in tumor growth was noted. Compared to the ɑ-human PSGL-1 clone KPL1, clone CHO131 used *in vivo* work performed less efficiently *in vitro* experiments, and thus, a choice of clone may be a contributing factor in the observed lowered efficiency of inhibition of tumor growth. Based on our *in vitro* findings from CyTOF experiments, a possible explanation for observed decline in tumor growth could be the inhibition of pro-survival pathways leading to a decrease in tumor cell proliferation. However, an effect on the tumor microenvironment cannot be ruled out at this time. Because of the potential redundancy in utilization of selectin ligands in neuroblastomas the most optimal route to inhibit signaling is to directly target selectins and not the ligand. Small-molecule inhibitors and blocking antibodies that interfere with selectin-ligand interactions are in development and are being tested in clinical trials [39]. Crizanlizumab, a humanized monoclonal antibody binding P-selectin [40] and blocking the interaction with PSGL-1, and presumably also with other selectin receptors, has been developed to manage pain crises resulting from microcirculatory vaso-occlusions that are prevalent in sickle cell patients. In a phase II trial with randomized sickle cell disease patients (n=198), crizanlizumab significantly lowered the incidence of pain crises. We speculate that this treatment could also help high risk neuroblastoma patients.

Most neuroblastomas express elevated levels of the disialoganglioside GD2, and antibodies against GD2 have been in research and development for over 20 years. Currently (March 2017) US National Library of Medicine clinical trials website lists 43 trials involving neuroblastoma treatment using GD2 immunotherapy in different forms and combinations with other therapies [41]. ɑ-GD2 antibodies also show limited effect against bulk neuroblastoma and different methods have been used to increase treatment efficacy. For example, several trials are in progress to enhance ɑ-GD2 efficacy with adoptive NK cell therapy as NK cells have been shown to be important in mediating the effects of ɑ-GD2 antibodies [42]. Dinutuximab, a chimeric human-mouse monoclonal antibody against GD2, was approved in 2015 to be used together with GM-CSF, IL2 and 13-cis retinoic acid to treat high risk neuroblastoma. Severe neuropathic pain due to expression of GD2 on peripheral nerves is one of the side effects of GD2 treatment. To address this ɑ-O-acetyl-GD2 antibodies have been developed [43, 44]. O-acetyl-GD2 derivative is expressed in neuroblastoma but not on peripheral nerves [43].

Tumor heterogeneity is a major problem in cancers. As our data show, there is not only considerable heterogeneity within the neuroblastoma cell population, but also their response to specific stimuli, as is seen with P-selectin in our case. For example, P-selectin increased levels of AKT as well as ERK and Src phosphorylation. Similarly, comparative analysis between L-, P- and E-selectin binding revealed generally comparable downstream survival signaling response. Results from Phase-1 trials of AKT inhibitor perifosine, which also inhibits ERK and JNK signaling pathways, have not yet been published [41, 45]. Another point of interest is the elevation in Notch2 and CD274 (PD-L1) expression in a subset of GD2-low tumor cells following P-selectin exposure. Since these cells are also CD133 positive, it is possible that they are tumor stem cells and may potentially exhibit therapeutic resistance to chemotherapy. The increased expression of CD274 (PD-L1) portends an additional resistance mechanism to immunotherapy in this sub population of tumor cells. Interestingly, its specific upregulation in P-selectin and not L- and E-selectin, suggest that any potential resistance mechanisms could be circumvented by blocking signaling through P-selectin binding. This hypothesis cannot be addressed in this study where an immunodeficiecnt model system has been employed. Thus, future work needs to address this possibility in the context of immunocompetent animal model systems.

In summary, our study is the first to address the importance of P-selectin, presumably emanating from the tumor microenvironment, to neuroblastoma pathology. The proteomic analyses described here also offer a methodology to not only address tumor heterogeneity and their variable response to treatment, but also identify a potential approach to target various sub populations within a tumor.

## MATERIALS AND METHODS

### Patient data mining

The Oncomine^™^ Platform (Thermo Fisher) and Oncogenomics Database were used for analysis and visualization of mRNA expression data from neuroblastoma patients [29] and to obtain Kaplan-Meier survival curves [30, 31].

### Tissue microarrays (TMA)

Based on approval from institutional review board paraffin-embedded neuroblastoma patient tumor samples were obtained as a TMA from the Children’s Oncology Group Neuroblastoma Biology Committee and the Biopathology Center (Columbus, OH, USA). Heat-mediated antigen retrieval was performed in citrate buffer, pH 6.0 and primary antibodies used were monoclonal ɑ-SELP (clone Psel.KO.2.5, LSBio) and ɑ-PSGL-1 (clone KPL1, EMD Millipore). Detailed staining protocol can be found in Supplementary Materials and Methods.

### Cell culture

Human neuroblastoma cell lines SK-N-BE(2), SK-N-AS, SK-N-SH, SH-SY5Y were purchased from American Type Culture Collection. SH-EP cells were provided by Susan Cohn (The University of Chicago Children’s Hospital) and NBL1 cells were isolated from bone marrow aspirate of metastatic neuroblastoma patient in MD Anderson Cancer Center. SK-N-BE(2) and NBL1 cells are NMYC amplified. All cells were STR DNA fingerprinted in Characterized Cell Line Core Facility at MD Anderson. Cells were cultured in standard conditions in RPMI 1640 supplemented with 10% FBS, 100 U/ml penicillin and 100 μg/ml streptomycin. In some experiments serum-free neural sphere formation supporting Gibco Neurobasal media was used, supplemented with 2% B27 (Gibco/Life Technologies), 20 ng/ml human bFGF (EMD Millipore), 20 ng/ml human EGF (Invitrogen), 1% glutamine and antibiotics as above.

### Flow analyses

Cells were detached with PBS and counted with Cellometer Vision CBA (Nexcelom Bioscience) imaging cytometer. Depending on experiment PBS containing 2% FBS either with or without Ca^2+^ and Mg^2+^ was used as suspension and wash buffer. Within an experiment the number of cells was adjusted to be equal in all samples. CD24 and GD2 APC (BioLegend) and CD44 PE (BD Pharmingen Biosciences) were used according to manufacturer’s instructions and surface staining was measured using a FACS Calibur or LSRFortessa (Becton Dickinson) flow cytometer and analyzed using FlowJo software (TreeStar). Monoclonal mouse α-human PSGL-1 (EMD Millipore, clone KPL1 and R&D Systems, clone CHO131) was detected by APC α-mouse Ig (BD Pharmingen Biosciences). For selectin binding assays human recombinant selectin-Fc chimera or control IgG Fc fragment (Jackson ImmunoResearch) at 10 μg/ml were incubated 30 min at room temperature (RT) with dissociated cells. After washing the cells APC-conjugated α-human IgG Fc (BioLegend) antibodies were added and incubated for 30 min. Bound selectins were measured as above. Recombinant human L-selectin, P-selectin and E-selectin Fc chimeras were purchased from R&D Systems.

### Gene knockout and silencing

CD24 Double Nickase and Control Double Nickase Plasmids (Santa Cruz Biotechnology) were transfected according to the manufacturer’s instructions using Fugene HD transfection reagent (Promega) into SH-EP cells. Two days after transfection GFP expressing cells were sorted, amplified and then exposed to puromycin (Clontech Laboratories) selection for seven days. Knockout cells are a mixture of mono- and biallelic *CD24* knockouts.

Lentiviruses for *PSGL-1* silencing were produced in MD Anderson shRNA and ORFeome Core from pGIPZ shRNA clones V3LHS_357201 and RHS4346 (GE Dharmacon). Neuroblastoma cells were plated on previous day and then transduced with lentivirus-polybrene (final 8 μg/ml) mixture. On the third day GFP expressing cells were sorted and then amplified. All sorting was done by Flow Cytometry and Cellular Imaging Core Facility at MD Anderson.

### Western blotting

Cells were either lysed directly or after incubation with P-selectin chimera or control Fc in PBS containing Ca^2+^, Mg^2+^ and 2% FBS. Cells were lysed on ice in a buffer containing 20 mM Tris pH 8, 137 mM NaCl, 10% glycerol, 1% Nonidet P-40 Substitute, 2 mM EDTA, supplemented with EDTA-free Protein and Phosphatase Inhibitor Cocktails (Roche). Supernatants were either used immediately or frozen. Protein concentrations were determined by BCA assay (Pierce). Samples were run on SDS-PAGE and transferred onto polyvinylidene fluoride membranes (BioRad). Membranes were incubated with rabbit α-p-Src family (Tyr416, Cell Signaling Technology), mouse α-PSGL-1 (clone KPL1, EMD Millipore) or rabbit α-CD24 (FL-80, sc-11406, Santa Cruz Biotechnology) antibodies followed by HRP-conjugated secondary antibodies (GE Healthcare). HRP conjugated β-actin was used as loading control (Abcam). Blots were developed using Pierce ECL Plus Western Blotting Substrate (Lumigen Inc.).

### Mass cytometry

Dissociated, selectin-Fc and Fc treated cells were fixed with methanol-free formaldehyde (Polysciences), stained with metal tagged antibodies provided by MD Anderson Flow Cytometry Core (staining protocol and table of antibodies used can be found in Supplementary Materials and Methods), and run on DVS CyTOF Mass Cytometer. Mass cytometry data files (.fcs) were first filtered using FlowJo to remove the normalization beads, doublets, and debris following a gating strategy shown in Supplementary Figure 4. Cleaned data was ported into R (version 3.2.1, The R Foundation for Statistical Computing) to generate the viSNE maps using the ‘Rtsne’ package [46]. The SPADE trees were generated using the SPADEv3.0 implemented in MATLAB R2014b [47]. Only surface markers were used as clustering parameters for both algorithms. Protein expression heat maps were based on cluster specific data exported from MATLAB and generated using the ‘ggplot2’ package in R. Some heat maps from viSNE run data were prepared using Cluster 2_5 and TreeView (Figures 4B and 5A).

### *In vitro* inhibition of P-selectin binding

Detached SK-N-BE(2) cells were either treated or mock treated with (0.3 U/ml) neuraminidase in Ca^2+^, Mg^2+^ PBS for 1 h at 37°C. Cells were washed, suspended into complete media and plated with 50 μM P-selectin inhibitor or DMSO (control). After two days P-selectin binding assays were performed. P-selectin inhibitor sc-220688 (2-Acetamido-4-fluoro-1,3,6-tri-O-acetyl-2,4-dideoxy-D-glucopyranose) was purchased from Santa Cruz Biotechnology and *Vibrio cholerae* neuraminidase from Sigma-Aldrich.

### Animal experiments

Animal experiments were approved by institutional IACUC committee (#00000449). Four million live SK-N-BE(2) cells were injected subcutaneously into the right flank of each NOD.Cg-*Prkdc*^*scid*^ *Il2rg*^*tm1Wjl*^ Tg(CMV-IL3, CSF2, KITLG)1Eav/MloySzJ (NSG-SGM3) mouse. At fourth week after injections mice began to show palpable tumors and were divided into three groups, weighted and treated as follows: control group received mouse IgG2a (BioLegend), second group received ɑ-human PSGL-1 (clone CHO131, R&D Systems) and the third group received Ultra-LEAF ɑ-mouse CD62P (clone RMP-1, BioLegend). All groups received treatment by intravenous (i.v.) tail vein injection once a week during a three week period the dosage being always 150 μg per 30 g of mouse. The size of the tumors and mouse weights were monitored constantly. Student’s two-tailed t-test was used to determine p-values. Paraffin sections of mouse tumors were stained with haematoxylin eosin (H&E) and after heat-mediated antigen retrieval by rat ɑ-mouse CD45 (clone 30-F11, Tonbo Biosciences) antibodies. Images were captured using Nikon NIS-Elements imaging software and CD45 positive cells were counted manually. Detailed staining protocol can be found in Supplementary Materials and Methods.

## SUPPLEMENTARY MATERIALS FIGURES AND TABLE



## Abbreviations

BSA: bovine serum albumin
CyTOF: mass cytometry
FBS: fetal bovine serum
H&E: haematoxylin and eosin
PSGL-1: P-selectin glycoprotein ligand-1
SDS-PAGE: sodium dodecyl sulfate polyacrylamide gel electrophoresis
