# Supplementary material for: Targeting P-selectin blocks neuroblastoma growth
Source: Oncotarget. 2017 Sep 28;8(49):86657–70. doi: 10.18632/oncotarget.21364 (PMC5689715; doi:10.18632/oncotarget.21364)
Supplement: Supplementary file 1 [file oncotarget-08-86657-s001.pdf]

Targeting P-selectin blocks neuroblastoma growth

SUPPLEMENTARY MATERIALS

Tissue microarrays (TMA)

Paraffin-embedded neuroblastoma patient tumor samples were obtained as a TMA from the Children's Oncology Group Neuroblastoma Biology Committee and the Biopathology Center in Columbus, OH based on approval from institutional review board. Slides were deparaffinized and rehydrated. Heat-mediated antigen retrieval was performed using a vegetable steamer (25 minutes in 0.01 M citrate buffer pH 6.0). Endogenous peroxidase was blocked with 3% hydrogen peroxide and slides were blocked with fish gelatin (Biotium). Primary antibodies were incubated for 45 min. Slides were washed and incubated with 4plus biotinylated goat α-mouse IgG (Biocare Medical) for 40 min, washed and again incubated with 4plus streptavidin-HRP (Biocare Medical) for 20 min. Immunoreaction was detected with stable diaminobenzidine (Invitrogen) and Gill #3 hematoxylin (Sigma-Aldrich) was used for background staining. Primary antibodies used were to mouse α-human P-selectin (LS-B3656, clone Psel.KO.2.5, LifeSpan BioSciences) and mouse α-human PSGL-1 (MAB4092, clone KPL1, EMD Millipore).

CD45 Immunohistochemistry

Sections of paraffin-embedded SK-N-BE(2) tumors in mice were initially treated as above. Slides were blocked with 5% goat serum for 1 h, followed by overnight incubation at +4°C with rat α-mouse CD45 (clone 30-F11, Tonbo Biosciences) antibodies at concentration of 1 μg/ml. After incubating with HRP α-rat antibodies (BioRad, 1:100) for 2 hrs the immunostaining was detected as above. Images were captured using inverted Nikon Eclipse Ti-E microscope and NIS-Elements imaging software.

Staining for CyTOF

Cells were washed twice and incubated in PBS, detached by trituration and suspended into PBS containing Ca^{2+}, Mg^{2+} and 2% FBS. Cells were filtered through 35 μm mesh, stained by Trypan Blue and counted using Cellometer Vision CBA (Nexcelom Bioscience) imaging cytometer. 1 million cells per sample were treated with Fc and E-, L- and P-selectin-Fc (both at 10 μg/ml) for 30 and 60 min at RT. Cells were washed with PBS containing Ca^{2+}, Mg^{2+} and fixed with 1.6% methanol-free formaldehyde (Polysciences) for 10 min at RT. After fixation samples were washed twice with 0.5% BSA (IgG-Free, Protease-Free BSA, Jackson ImmunoResearch) in PBS and stored at +4°C overnight. Staining protocol was provided by MD Anderson Flow Cytometry Core. Briefly, cells were washed with 0.5% BSA in PBS and incubated 30 min at RT with antibodies against surface markers. Cells were washed once in above buffer and stained with cisplatin (Cell-ID™ Cisplatin, Fluidigm), even though cells were already fixed. Samples were washed thrice with 0.5% BSA in PBS and then permeabilized with cold methanol, washed again twice and incubated with antibodies against intracellular proteins for 1h at RT. Samples were washed thrice with 0.5% BSA in PBS and stained by 100 nM Ir-intercalator (Cell-ID™ Intercalator-Ir, Fluidigm), 1.6% formaldehyde in PBS overnight. On the following day samples were washed twice with 0.5% BSA in PBS, suspended in 0.1% BSA in water, filtered through 35 μm mesh and counted. Depending on sample cell recovery was between 500 000 and 800 000 cells.

Supplementary Table 1: Antibodies used in CyTOF

Target of antibody	Label	Clone	Source	Catalog #
ERK1	139La	250603	R&D Systems	MAB1940
FAK	141Pr	D2R2E	Cell Signaling Technology	13009
AKT	143Nd	302407	R&D Systems	MAB17751
p-Tyrosine	144Nd	p-Tyr-100	DVS-Fluidigm	3144003A
p-STAT5(Y694)	147Sm	47	DVS-Fluidigm	3147012A
p-SYK	149Sm	1120-722	BD	558167
CD274, PD-L1	150Nd	29E.2A3	BioLegend	329702
Survivin	151Eu	Polyclonal	R&D Systems	AF6471
GD2	152Sm	14.G2a	BD	554272
CD133/2	153Eu	293C3	Miltenyi	130-090-851
Src	155Gd	36D10	Cell Signaling Technology	2109
P-p38(180/182)	156Gd	D3F9	DVS-Fluidigm	3156002A
p-AKT	159Tb		DVS-Fluidigm	
p-PI3K	160Gd	polyclonal	Abcam	ab61801
CD54, ICAM-1	162Dy	HCD54	BioLegend	322702
c-Myc	163Dy	D84C12	Cell Signaling Technology	5605
p-Src(Tyr416)	164Dy	D49G4	Cell Signaling Technology	6943
p53	165Ho	184721	R&D Systems	MAB1355
CD44	166Er	BJ18	DVS-Fluidigm	3166001B
p-ERK1/2	167Er	D13.14.4E	DVS-Fluidigm	3167005A
Ki67	168Er	(Ki67) X8	DVS-Fluidigm	3168001B
CD24	169Tm	ML5	DVS-Fluidigm	3169004B
APC	170Er		BD	
Notch3	171Yb	MHN3-21	BioLegend	345407
CD273, PD-L2	172Yb	24F.10C12	DVS-Fluidigm	3172014B
Notch2	174Yb	235-162A	Riitta Nolo	
p-FAK(Y397)	175Lu	D20B1	Cell Signaling Technology	8556
CD56	176Yb	CMSSB	DVS-Fluidigm	3176003B

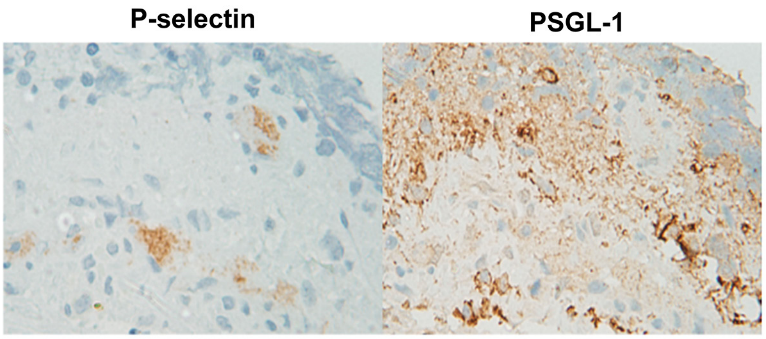

Supplementary Figure 1: P-selectin and PSGL-1 staining of stage 4 neuroblastoma.

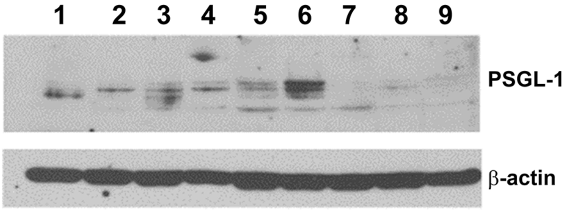

Supplementary Figure 2: Western analysis of primary tumor samples showing the high variability of PSGL-1 expression.

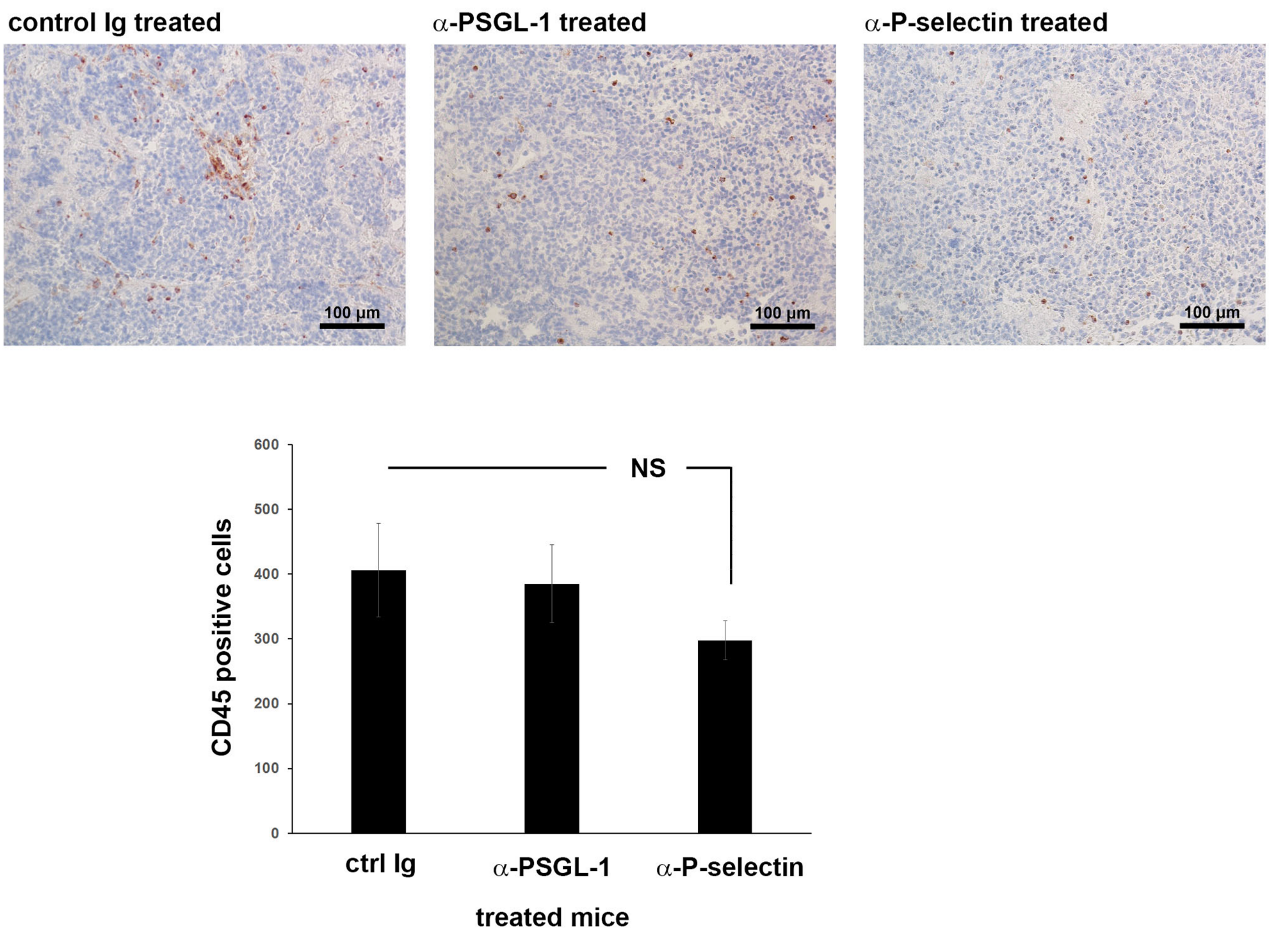

Supplementary Figure 3: Immune cell infiltration in mouse SK-N-BE(2) tumors is revealed by CD45 staining. In each treatment group n = 3. CD45 positive cells from six viewing areas/mouse were counted. The difference in numbers of CD45 positive cells between groups was not statistically significant.

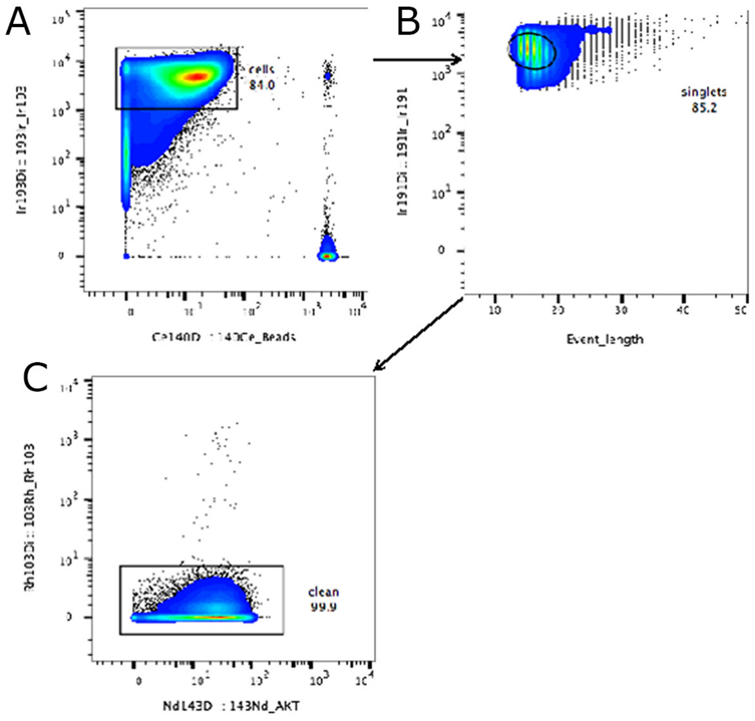

Supplementary Figure 4: CyTOF gating strategy. (A) To gate out beads, the iridium 193 channel is plotted against the bead channel to identify events with high iridium uptake and low bead counts. **(B)** To remove doublets and debris, iridium is further plotted against event length and events are gated on the bright population with short event length and high iridium uptake. Dual high iridium uptake and longer event lengths likely represent doublets, while short event length with less iridium may represent debris. **(C)** Debris is further out by plotting an empty channel (in this case, Rh103Di) against any relevant channel. Events with high values in a channel where there should be no metal detection are debris and removed before downstream analyses.
